# Remarkably Low *KIR* and *HLA* Diversity in Amerindians Reveals Signatures of Strong Purifying Selection Shaping the Centromeric *KIR* Region

**DOI:** 10.1093/molbev/msab298

**Published:** 2021-10-11

**Authors:** Luciana de Brito Vargas, Marcia H Beltrame, Brenda Ho, Wesley M Marin, Ravi Dandekar, Gonzalo Montero-Martín, Marcelo A Fernández-Viña, A Magdalena Hurtado, Kim R Hill, Luiza T Tsuneto, Mara H Hutz, Francisco M Salzano, Maria Luiza Petzl-Erler, Jill A Hollenbach, Danillo G Augusto

**Affiliations:** 1 Programa de Pós-Graduação em Genética, Departamento de Genética, Universidade Federal do Paraná, Curitiba, PR, Brazil; 2 Department of Neurology, Weill Institute for Neurosciences, University of California, San Francisco, San Francisco, CA, USA; 3 Department of Pathology, Stanford University School of Medicine, Palo Alto, CA, USA; 4 School of Human Evolution and Social Change, Arizona State University, Tempe, AZ, USA; 5 Departamento de Análises Clínicas, Universidade Estadual de Maringá, Maringá, PR, Brazil; 6 Departamento de Genética, Instituto de Biociências, Universidade Federal do Rio Grande do Sul, Porto Alegre, RS, Brazil; 7 Department of Epidemiology and Biostatistics, University of California, San Francisco, San Francisco, CA, USA

**Keywords:** killer-cell immunoglobulin-like receptor, high resolution, population, evolution, human leukocyte antigen

## Abstract

The killer-cell immunoglobulin-like receptors (KIR) recognize human leukocyte antigen (HLA) molecules to regulate the cytotoxic and inflammatory responses of natural killer cells. *KIR* genes are encoded by a rapidly evolving gene family on chromosome 19 and present an unusual variation of presence and absence of genes and high allelic diversity. Although many studies have associated *KIR* polymorphism with susceptibility to several diseases over the last decades, the high-resolution allele-level haplotypes have only recently started to be described in populations. Here, we use a highly innovative custom next-generation sequencing method that provides a state-of-art characterization of *KIR* and *HLA* diversity in 706 individuals from eight unique South American populations: five Amerindian populations from Brazil (three Guarani and two Kaingang); one Amerindian population from Paraguay (Aché); and two urban populations from Southern Brazil (European and Japanese descendants from Curitiba). For the first time, we describe complete high-resolution *KIR* haplotypes in South American populations, exploring copy number, linkage disequilibrium, and KIR–HLA interactions. We show that all Amerindians analyzed to date exhibit the lowest numbers of KIR–HLA interactions among all described worldwide populations, and that 83–97% of their KIR–HLA interactions rely on a few HLA-C molecules. Using multiple approaches, we found signatures of strong purifying selection on the *KIR* centromeric region, which codes for the strongest NK cell educator receptors, possibly driven by the limited HLA diversity in these populations. Our study expands the current knowledge of *KIR* genetic diversity in populations to understand *KIR–HLA* coevolution and its impact on human health and survival.

## Introduction

Natural killer (NK) cells are cytotoxic lymphocytes that were first discovered due to their ability to spontaneously kill tumor cells in vitro without prior sensitization (Herberman and Holden 1978) and later recognized as critical components of the first line of defense against tumor and infected cells (Morvan and Lanier 2016; Flórez-Álvarez et al. 2018). Among the receptors that control NK cell cytotoxicity is the killer-cell immunoglobulin-like receptor (KIR) family, which recognizes human leukocyte antigen (HLA) molecules as primary ligands (Lanier and Phillips 1995; Moretta et al. 1996).

KIR molecules are encoded by a highly polymorphic gene family located on the chromosome region 19q13.4, characterized by an uncommon and complex structural variation of presence and absence of genes (Wende et al. 1999; Wilson et al. 2000). The homology and high sequence similarity among the 13 *KIR* loci contribute to the occurrence of nonreciprocal recombination (Wilson et al. 2000; Martin et al. 2003), which generates duplication and deletion of entire genes or groups of genes and the formation of hybrid genes and alleles (Martin et al. 2003; Norman et al. 2009; Traherne et al. 2010; Roe et al. 2017). The *KIR* region is known for its complexity and rapid evolution (Khakoo et al. 2000; Sambrook et al. 2005; Guethlein et al. 2007).

The presence and absence of genes generate a wide diversity of *KIR* gene-content haplotypes, classified into two groups: *A* and *B* (Uhrberg et al. 2002). Both *KIR A* and *B* haplotypes are present in all studied human populations; however, their frequencies vary significantly. For example, haplotype *A* is present in approximately 80% of Japanese individuals (Yawata et al. 2006), but only 2% of Australian aborigines (Toneva et al. 2001). Despite a large number of known haplotypes among human populations (Hsu et al. 2002; Uhrberg et al. 2002; Martin et al. 2008; Pyo et al. 2013; Roe et al. 2017), most haplotypes are the result of different combinations of a smaller set of centromeric (flanked by *KIR3DL3* and *KIR3DP1*) and telomeric segments (flanked by *KIR2DL4* and *KIR3DL2*) (Pyo et al. 2010; Hollenbach et al. 2012). This feature is most likely driven by a hotspot facilitating the recombination of telomeric and centromeric segments (Traherne et al. 2010).

In contrast with *KIR* genes, which emerged 23–1.7 Ma (Wilson et al. 2000; Pyo et al. 2010; Parham and Guethlein 2018), the *HLA* constitutes an evolutionary older gene family that arose 49–22 Ma (Piontkivska and Nei 2003; Fukami-Kobayashi et al. 2005). *HLA* genes are located within the major histocompatibility complex on chromosome 6 (Horton et al. 2004) and are the most polymorphic genes in the human genome (Hill 1999). The interaction of KIR and HLA is critical for NK cell education during the early stages of maturation (Kärre et al. 1986; Kim et al. 2005), for regulating NK cell cytotoxicity (Smyth et al. 2005; Lanier 2008), and reproduction (Hiby et al. 2004; Xiong et al. 2013; Blokhuis et al. 2017). In addition, combinations of *KIR–HLA* have been associated with numerous diseases (Martin et al. 2002; Van der Slik et al. 2003; Khakoo et al. 2004; Nelson et al. 2004; Carrington et al. 2005; Augusto et al. 2012; Augusto 2016; Anderson et al. 2020), and there is growing evidence that these two families coevolve as a unique system (Gendzekhadze et al. 2009; Norman et al. 2013; Augusto and Petzl-Erler 2015; Moffett and Colucci 2015; Vargas et al. 2020).

Despite the relevance of *KIR* for disease and survival, the complexity of the structural variation of haplotypes and the high sequence similarity among genes impose technical difficulties on their study. Although over a thousand *KIR* alleles have been deposited in the IPD (ImmunoPolymorphism Database)-KIR (Robinson et al. 2015), the distribution of these alleles in global populations is poorly known. To date, the vast majority of population genetics studies have only analyzed *KIR* at the gene-content level and sometimes in combination with a few HLA ligands (González-Galarza et al. 2015). The study of allelic diversity at high resolution for all *KIR* genes is still restricted to a few populations (Norman et al. 2016; Nemat-Gorgani et al. 2018, 2019; Alicata et al. 2020; Solloch et al. 2020; Amorim et al. 2021; Deng et al. 2021; Tao et al. 2021).

Our previous work and that of others have shown a limited number of *KIR* gene-content haplotypes in Amerindians (Gutiérrez-Rodríguez et al. 2006; Flores et al. 2007; Single et al. 2007; Augusto et al. 2015). However, *KIR* allelic diversity has only been described for a single Amerindian population, the Yucpa from Venezuela, with high-resolution genotyping restricted to a few loci (Gendzekhadze et al. 2009). Evidence has shown that Eastern Amerindians, such as those living in Brazil, bear even lower genetic diversity than Western Amerindians (Wang et al. 2007). Additionally, previous remarkable studies identified several high-frequency *HLA* alleles in Brazilian Amerindians never found in Amerindians from North America or any other global population (Belich et al. 1992; Watkins et al. 1992; Parham et al. 1997).

Here, we present the first high-resolution characterization of *KIR* allelic variation in seven South American populations from Brazil and one from Paraguay. We analyzed six isolated Amerindian populations from Guarani, Kaingang, and Aché ethnicities, and also individuals of European and Japanese ancestries living in Southern Brazil. We deliver the first study to analyze the in-depth *KIR* diversity at high resolution in South American populations, identifying signatures of purifying selection on the centromeric *KIR* region, possibly driven by the reduced diversity of HLA ligands.

## Results

### Amerindians from Our Study Exhibit the Lowest *KIR* Diversity among All Worldwide Populations Analyzed to Date

**Fig. 1.**
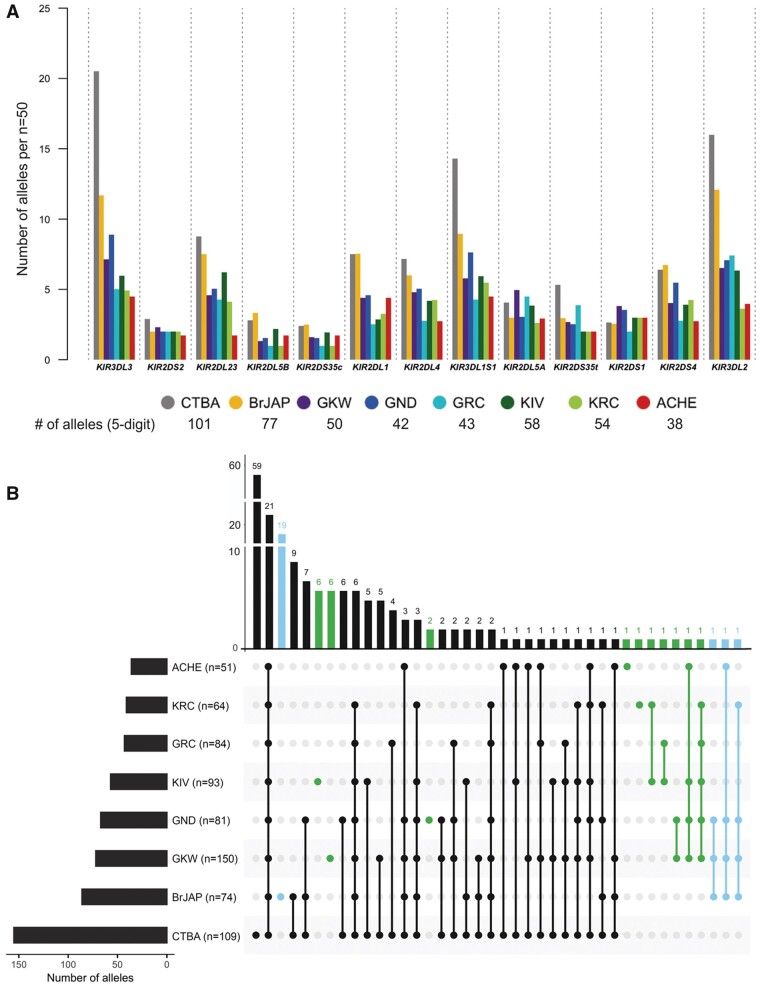
Overview of the *KIR* diversity in the study populations. (*A*) The bar plot shows the number of high-resolution (five-digit) alleles found for each *KIR* gene in the study populations in random subsets of *n* = 50. (*B)* Allele sharing among study populations. The UpSetR plot is an alternative to the Venn diagram and allows better visualization when comparing multiple sets (populations). The vertical bars on the upper portion represent the absolute number of shared alleles by a unique intersect of populations. On the bottom, filled circles indicate the populations of each intersect that share the number of alleles represented by the vertical bars. For example, the first vertical bar indicates that 59 *KIR* alleles were found exclusively in CTBA (only filled circle), whereas the second vertical bar indicates that 21 alleles are shared by all populations (all circles are filled). The horizontal bars, in the left, represent the total number of alleles found in each population. Sets of alleles found or shared exclusively in Amerindians are highlighted in green, and in blue are the sets of alleles found or shared only in Japanese or in Japanese and Amerindians. The study populations include Brazilians of European ancestry (CTBA); Brazilians of Japanese ancestry (BrJAP); Aché (ACHE); GKW; GND; GRC; KIV; and KRC.

We define as a *KIR* allele each unique DNA sequence at a particular locus. To directly compare the allelic diversity of the study populations, we randomly selected 50 individuals from each study population. We observed a remarkably low diversity of *KIR* alleles in the Amerindian study populations, averaging 53 ± 9 alleles at five-digit resolution per population. The number of *KIR* alleles was especially small in Aché (ACHE; 41 alleles per *n* = 50), Kaingang from Rio das Cobras (KRC, 46 alleles per *n* = 50), and Guarani Mbya (GRC; 48 alleles per *n* = 50). In sharp contrast, we observed 123 and 89 alleles per *n* = 50 in the two Brazilian urban populations, European and Japanese descendants, respectively. Individually, *KIR3DL3* and *KIR3DL2* were the two genes with the highest number of alleles (fig. 1*A*). The complete list of allelic frequencies is described in supplementary table 1, Supplementary Material online.

Multiple alleles observed in our Amerindian samples were not found in the two urban populations. Six of these alleles observed exclusively in Amerindians were found only in Kaingang from Ivaí (KIV), and six were observed only in Guarani Kaiowá (GKW) (fig. 1*B* and supplementary table 2, Supplementary Material online). Overall, the six Amerindian populations shared an average of 76.64% of their *KIR* alleles. A high proportion of alleles is shared between Amerindians and Japanese descendants (66.93%), and there is reduced proximity between Euro-descendants and Amerindians, only 60.11% (*P *<* *0.001; supplementary table 3, Supplementary Material online).

**Fig. 2.**
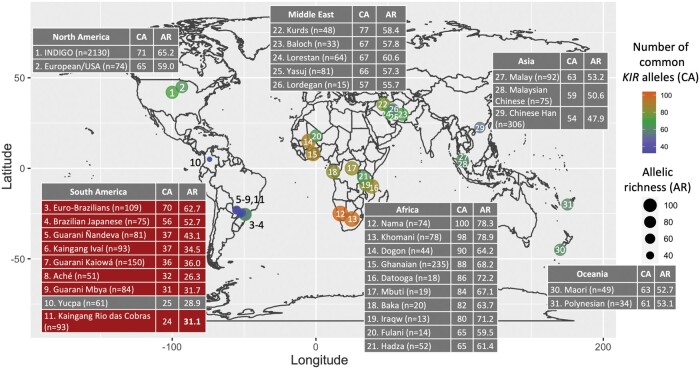
Amerindians exhibit the lowest *KIR* diversity among worldwide populations. Tables display the number of *KIR* alleles at three-digit resolution with frequencies greater or equal to 1% (common alleles, CA) and allele richness (AR) in global populations. Red boxes indicate analyzed in the present study. The complete data set is given in supplementary table 4, Supplementary Material online. Raw data from worldwide populations were obtained from previous studies (Gendzekhadze et al. 2009; Vierra-Green et al. 2012; Norman et al. 2013; Nemat-Gorgani et al. 2014, 2018, 2019; Alicata et al. 2020; Amorim et al. 2021; Deng et al. 2021; Tao et al. 2021). INDIGO = European Americans and healthy controls from the Immunogenetics of Neurological DIseases working GrOup (Amorim et al. 2021).

According to the *KIR* official nomenclature, three-digit resolution means the identification of the DNA sequence of each allele that allows the distinction only of the substitutions that change the protein sequence (Robinson et al. 2015). We define as an allotype every unique protein encoded by a *KIR* allele. To maximize the comparison of *KIR* diversity and include multiple global populations (Jones et al. 2006; Gendzekhadze et al. 2009; Vierra-Green et al. 2012; Norman et al. 2013; Nemat-Gorgani et al. 2014, 2018, 2019; Alicata et al. 2020; Amorim et al. 2021; Deng et al. 2021; Tao et al. 2021), we compared the study populations with others using *KIR* alleles at the three-digit resolution, which define the allotypes, considering only those with frequencies greater than or equal to 1%. With only 24, KRC has the lowest number of common (*f *≥ 1%) *KIR* three-digit alleles ever reported. The average number of common *KIR* alleles at three-digit resolution in Amerindians was 32 ± 5, whereas the other worldwide populations had an average of 73 ± 13 (fig. 2). We also estimated the allele richness for each population (fig. 2 and supplementary table 4, Supplementary Material online). We show a reduced *KIR* allele richness in the Amerindians compared with worldwide populations, also showing that Aché exhibited the lowest allelic richness to date. In addition, we found a strong correlation (*r* = 0.97; *P *=* *1.5 × 10^−18^) between number of common alleles and allele richness (supplementary fig. 1*A*, Supplementary Material online) and no correlation between sample sizes and number of common alleles (supplementary fig. 1*B*, Supplementary Material online).

**Fig. 3.**
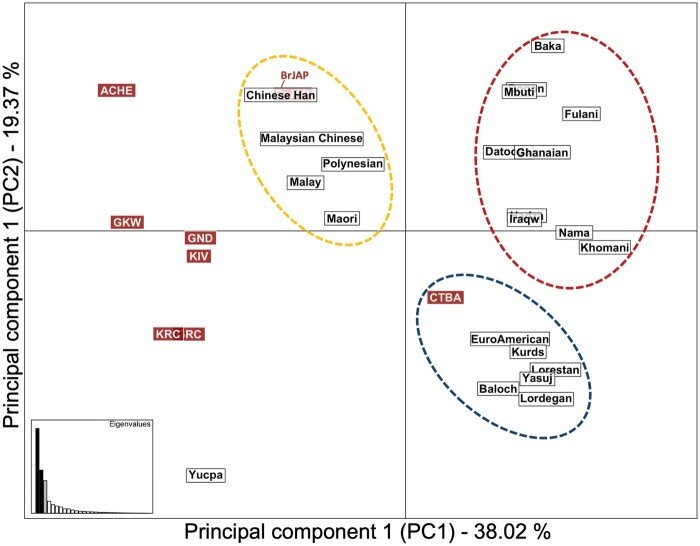
Principal components analysis (PCA) of *KIR* variation. In PCA, data containing allele frequencies of all *KIR* genes are reassigned to new variables, called principal components, that summarize the total distribution of the variation. The PCA included worldwide populations studied at the allele level for all *KIR* genes. To maximize our analysis, we included studies that described *KIR* variation at three-digit allelic resolution. The rectangles resume *KIR* diversity in each population. The two first components plotted here explain 57.39% of *KIR* variation. The dashed circles highlight the similarity of *KIR* diversity shared by a group of populations; yellow: Southeast Asians; red: Africans; blue: European-descendants and Middle-Easterns. The study populations include Brazilians of European ancestry (CTBA); Brazilians of Japanese ancestry (BrJAP); Aché (ACHE); GKW; GND; GRC; KIV; and KRC. Data from previously analyzed populations are found in previous studies (Gendzekhadze et al. 2009; Vierra-Green et al. 2012; Norman et al. 2013; Nemat-Gorgani et al. 2014, 2018, 2019; Alicata et al. 2020; Amorim et al. 2021; Deng et al. 2021; Tao et al. 2021).

The study populations exhibit an overall great population differentiation (supplementary fig. 2, Supplementary Material online). The genes responsible for most of the differentiation were *KIR2DL1* (median *F*_ST_ = 0.11), *KIR2DL23* (median *F*_ST_ = 0.10), and *KIR3DL1S1* (median *F*_ST_ = 0.09; supplementary fig. 2*C*, Supplementary Material online). We generated bidimensional plots using the principal component analysis (PCA) to visualize the overall difference in allele frequencies across all *KIR* loci in populations. Populations formed three clusters (fig. 3): in red, all Africans; in yellow, East Asians (including BrJAP) and Oceanic populations; in blue, all Middle Eastern and Euro-descendant populations. The Amerindians did not form a cluster despite being separated from all other populations. However, when the *KIR* centromeric and telomeric were plotted separately, we observed greater proximity for Amerindian groups in telomeric than centromeric *KIR* genes (supplementary fig. 3, Supplementary Material online).

### Remarkably High Frequencies of *cA01* Haplotypes in Amerindians: Carrier Frequency Reaches 100% in Aché

**Fig. 4.**
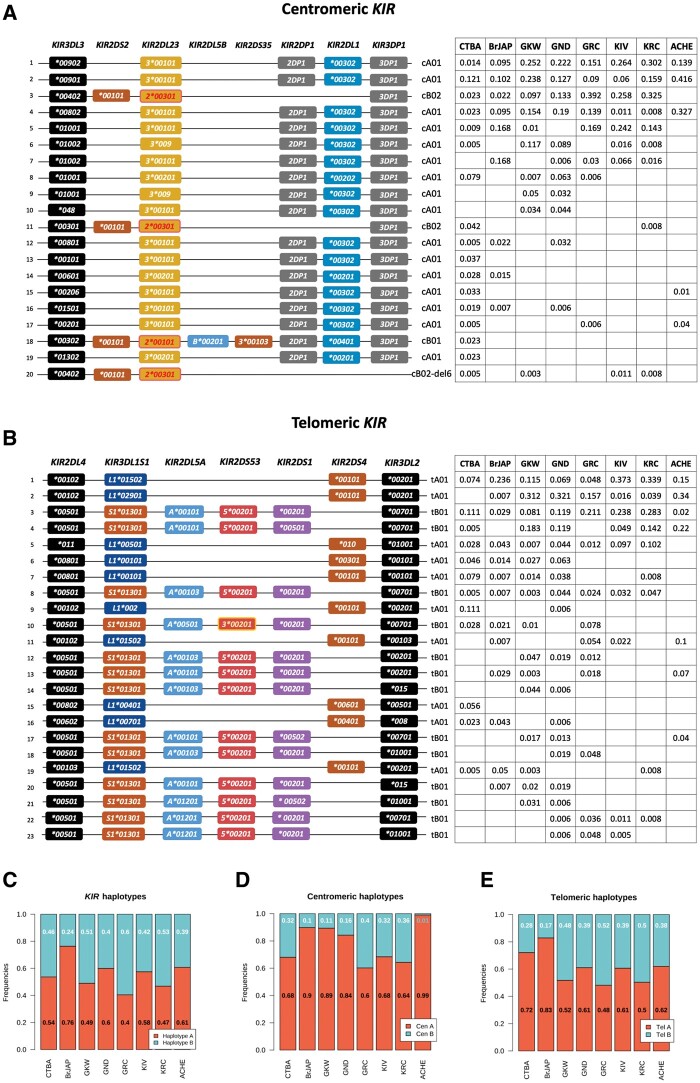
Common high-resolution *KIR* allelic haplotypes in the study populations. (*A*) Centromeric and (*B*) telomeric haplotypes are shown. The classification of each haplotype is indicated on the right side of each haplotype, followed by a box containing their relative frequencies. (*C*) Bar plot shows the proportion of *KIR A* and *B* haplotypes in the eight South American study populations. These are subclassified according to the gene content of (*D*) centromeric (*Cen A* and *Cen B*) and (*E*) telomeric (*Tel A* and *Tel B*) regions. The study populations include Brazilians of European ancestry (CTBA); Brazilians of Japanese ancestry (BrJAP); Aché (ACHE); GKW; GND; GRC; KIV; and KRC. Refer to supplementary tables 5 and 6, Supplementary Material online for the lists of all haplotypes.

For the first time, we report the frequencies of centromeric and telomeric haplotypes for Amerindians at both gene-content and high-resolution allelic levels (fig. 4*A* and *B* and supplementary tables 5 and 6, Supplementary Material online). We also provide pairwise linkage disequilibrium (LD) between high-resolution alleles in supplementary figures 4–11 and table 7, Supplementary Material online. The frequencies of *CenA* haplotypes were remarkably high in the Aché and two Guarani groups (fig. 4*D*). Specifically, *cA01* was present in 100% of ACHE individuals and exhibited frequencies ranging from 81.1% to 88.7% in GND (Guarani Ñandeva), GKW, and BrJAP (Brazilian Japanese) (supplementary table 8, Supplementary Material online). High frequencies of the *Cen A* haplotype were also observed in Asians, ranging from 76.7% to 93.2% (Yawata et al. 2006; Deng et al. 2021; Tao et al. 2021). In contrast, *Cen A* frequencies in other worldwide populations range from 29.0% to 69.4% in Africans (Nemat-Gorgani et al. 2018; 2019) and 65% to 69.0% in Iranians and European descendants (Vierra-Green et al. 2012; Alicata et al. 2020). Interestingly, *cB02* was highly frequent in GRC, carried by 39.3% of the population. As expected, greater diversity of haplotypes was observed in populations of European and Japanese ancestries, with 30 and 15 centromeric and 25 and 22 telomeric haplotypes at the gene-content level, respectively.

The most common gene content haplotype in all populations is *cA01∼tA01*, followed by *cA01∼tB01* or *cB02∼tB01* (supplementary table 8, Supplementary Material online). We identified haplotypes carrying large structural deletions or duplications involving multiple loci. The haplotype *cB02∼tB01-del6*, with the deletion of *KIR3DP1∼ KIR2DL4∼ KIR3DS1*, was found in six populations, with frequencies greater than 2% in Japanese descendants and 1% in Kaingang Ivaí (KIV). On the other hand, the genes deleted in *del6* are duplicated in the expanded haplotype *cA01∼tA01-ins4*.

Considering the common *KIR* haplotypes at high resolution found in at least three individuals, we observed 46 telomeric and 37 centromeric haplotypes at the allelic level. Among the less frequent allele-level haplotypes, we observed 119 centromeric 141 telomeric haplotypes found in less than three individuals, and only 0.67% of haplotypes were not resolved. The low-frequency haplotypes are usually variations of the most common ones, differing by only a few alleles in some specific genes. Allelic variation of *KIR3DL3* was responsible for around 30% of the less common centromeric haplotypes. This fact is likely the result of a recombination hotspot in *KIR3DL3* exon 5, which increases sequence variation in the gene and leads to lower LD with the remainder of the *KIR* centromeric region (Jones et al. 2006; Abi-Rached et al. 2010). On the telomeric region, the allelic variation on *KIR3DL1S1* differentiated most of the less frequent haplotypes. Overall, we found greater diversity on telomeric when compared with centromeric haplotypes for all Amerindian populations.

### HLA-C Ligands Almost Exclusively Mediate the KIR Regulation in All Amerindians Studied to Date

We provide a detailed analysis of the combinatorial diversity of KIR–HLA interactions in the study populations (table 1). Our study is the first to report the frequencies of HLA ligands in Aché. Like the other Amerindians in this study and the literature (Johnson et al. 1978; Petzl-Erler et al. 1993; Fernández-Viña et al. 1997; Parham et al. 1997; Layrisse et al. 2001; García-Ortiz et al. 2006), Aché also exhibited the complete lack of HLA-A3 and A11 ligands (supplementary table 9, Supplementary Material online). Interestingly, the Aché also exhibited a low frequency of Bw4 (*f* = 0.05), resulting in most of their KIR–HLA interactions being dependent on HLA-C.

**Table 1.** Functional KIR–HLA Interactions in the Study Populations.

HLA Epitope	KIR	CTBA	BrJAP	KIV	KRC	GND	GKW	ACHE	GRC
Bw4 (HLA-A)	KIR3DL1	0.09	0.17	0.02	0.01	0.05	0.01	0.02	
Bw4 (HLA-B)	KIR3DL1	0.16	0.16	0.13	0.10	0.04	0.05	0.01	0.04
A3	KIR3DL2	0.06	0.01		0.01				
A11	KIR3DL2	0.03	0.07		0.02	0.01	0.00		
A11	KIR2DS4	0.02	0.05		0.01	0.01			
C2	KIR2DL1	0.15	0.04	0.16	0.14	0.21	0.19	0.26	0.06
C1C2	KIR2DL2*	0.15	0.04	0.19	0.23	0.12	0.10	0.01	0.36
C1	KIR2DL3*	0.18	0.36	0.19	0.22	0.32	0.41	0.30	0.41
C2	KIR2DS1	0.06	0.01	0.11	0.13	0.14	0.16	0.17	0.09
C16	KIR2DS2	0.01					0.00		
C2	KIR2DS5*								
HLA-C*	KIR2DS4	0.10	0.09	0.20	0.13	0.09	0.06	0.22	0.05
Activating (%)		0.18	0.15	0.31	0.27	0.23	0.23	0.40	0.14
Inhibitory (%)		0.82	0.85	0.69	0.73	0.77	0.77	0.60	0.86
Mean KIR–HLA interactions per individual		6.92	5.83	5.03	4.71	4.32	3.40	2.96	2.86
(min–max)		(1–13)	(2–12)	(1–10)	(1–10)	(1–12)	(1–7)	(1–7)	(1–8)

Note.—Interaction values are given as the percentage of individuals that present that functional interaction in a population. Blank cells indicate that the interaction was not found in a population. Asterisks indicate that only a subset of the molecules is considered in the interaction, as detailed in the Materials and Methods section. The proportion of activating and inhibitory interactions in each population is also shown. Mean KIR–HLA indicates how many functional pairs one individual of each population has on average. The minimum and the maximum numbers of interactions observed in a single individual are shown in parenthesis. CTBA, Brazilians of European ancestry; BrJAP, Brazilians of Japanese ancestry; ACHE, Aché; GKW, Guarani Kaiowá; GND, Guarani Ñandeva; GRC, Guarani Mbya; KIV, Kaingang from Ivaí; KRC, Kaingang from Rio das Cobras.

**Fig. 5.**
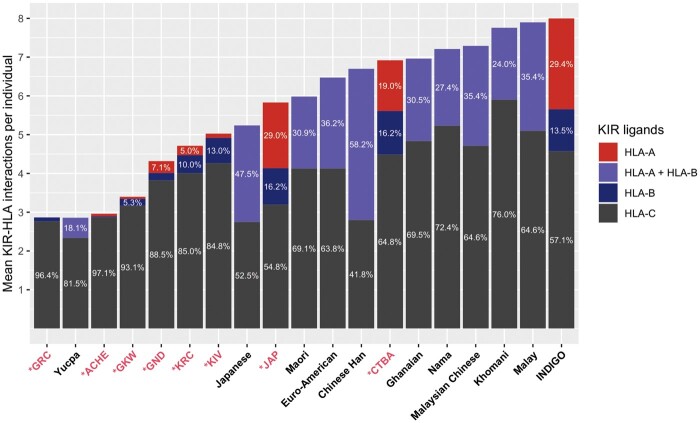
HLA-C ligands represent 85–97% of the total HLA ligands in the Amerindians from this study. Vertical bars represent the average of functional KIR–HLA interactions per individual in worldwide populations. Color code represents the proportion of these interactions that are represented by different HLA subgroups. *Populations from this study (in red) include GRC; Aché (ACHE); GKW; GND; KRC; KIV; Brazilians of Japanese ancestry (BrJAP); and Brazilians of European ancestry (CTBA). Data from previously analyzed populations are found in previous studies (Yawata et al. 2006; Gendzekhadze et al. 2009; Vierra-Green et al. 2012; Norman et al. 2013; Nemat-Gorgani et al. 2014, 2018; Amorim et al. 2021; Deng et al. 2021; Tao et al. 2021). INDIGO = European Americans and healthy controls from the Immunogenetics of Neurological DIseases working GrOup (Amorim et al. 2021).

On average, Amerindians in this study exhibited only ∼3 to 5 KIR–HLA interactions per individual (table 1). In contrast, the Japanese had an average of 5.8 KIR–HLA interactions. In the European-Brazilians, we found an average of 6.9. Among all interactions, HLA-C was responsible for 51.3% of the observed KIR–HLA interactions in Japanese and 57.8% in Euro-Brazilians. However, HLA-C has a remarkably high contribution to KIR–HLA interactions in Amerindians, ranging from 82.6% to 97.1% (fig. 5 and table 1; and supplementary table 10, Supplementary Material online). Moreover, more than 60% of the KIR–HLA interactions observed in all study populations are inhibitory, with *KIR2DL23* accounting for most of the considered interactions, ranging from 17.8% in Euro-Brazilians to 41.4% in GKW.

### Reduced *HLA* Diversity Imposes Purifying Selection on the Centromeric *KIR* Region

**Fig. 6.**
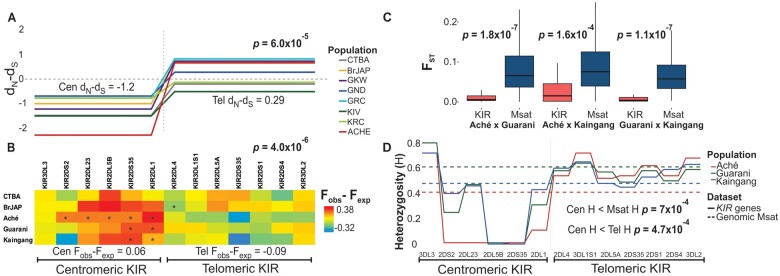
Signatures of purifying selection in the centromeric KIR region. (*A*) Analysis of nonsynonymous (d_*N*_) and synonymous (d_*S*_) substitutions in *KIR* genes. The rates of nonsynonymous substitutions are significantly reduced in the centromeric *KIR* region compared with the telomeric *KIR* (Cen d_*N*_–d_*S*_ < Tel d_*N*_–d_*S*_; *P *=* *6.0 × $10_{)}^{-5}$. The difference was calculated across the coding sequences of all *KIR* genes within the centromeric (*KIR3DL3∼KIR2DL1*) and telomeric (*KIR2DL4∼KIR3DL2*) regions. Positive values (d_*N*_ > d_*S*_) indicate positive selection and negative values (d_*N*_ < d_*S*_) indicate purifying selection. The study populations include Brazilians of European ancestry (CTBA); Brazilians of Japanese ancestry (BrJAP); Aché (ACHE); GKW; GND; GRC; KIV; and KRC. (*B*) Ewens–Watterson test of neutrality in all *KIR* genes. The color scale represents the differences between the observed homozygosity (*F*_obs_) and expected homozygosity (*F*_exp_). Asterisk indicates significative *P* values (*P *<* *0.025 or *P *>* *0.975). Shades of red in the centromeric region, combined with significant *P* values, indicate that homozygosity was observed at higher rates than expected under neutral evolution. The homozygosity rates were overall significantly reduced in the centromeric *KIR* region compared with the telomeric (Cen *F*_obs_–*F*_exp_ < Tel *F*_obs_–*F*_exp_; *P *=* *4.0 × $10_{)}^{-6}$. (*C*) The fixation index (*F*_ST_) analysis of the *KIR* allelic diversity in Amerindians compared with 678 genomic microsatellite markers (Msat) shows that differentiation is significantly reduced in *KIR*. (*D*) Compared with genomic markers, the heterozygosity (H) in the centromeric *KIR* region is reduced in Aché, Guarani, and Kaingang (*P *=* *7.0 × 10^−4^), whereas the telomeric has similar heterozygosity rates to the genomic microsatellite markers (*P *=* *0.12). The differences in heterozygosity rates between centromeric and telomeric *KIR* are highly significant (Cen *H* < Tel *H*; *P *=* *4.7 × 10^−4^).

Based on the observation of a small number of HLA ligands in the Amerindians, we hypothesized that reduced *HLA* diversity could be shaping the *KIR* diversity in these populations. To test this hypothesis, we first calculated the difference of synonymous to nonsynonymous substitution (d_*N*__–_d_*S*_) for all *KIR* loci (fig. 6*A*). We observed a significant reduced nonsynonymous rate in the centromeric region (Cen d_*N*__–_d_*S*_ = −1.2) in comparison with the telomeric region (Tel d_*N*__–_d_S_ = 0.29) when we analyzed all study populations (*P *=* *6 × 10^−5^). Individually, the deviation of neutrality in the *KIR* centromeric region was significant in the Aché (d_*N*__–_d_*S*_ = −2.28, *P *=* *0.02), the population with the most limited diversity of HLA allotypes and with 97.1% of the KIR–HLA interactions mediated by HLA-C alone. The negative values in the centromeric *KIR* indicate an excess of synonymous substitutions in lieu of nonsynonymous substitutions, which could indicate purifying selection.

Next, we applied the Ewens–Watterson test of neutrality. Although the d_*N*__–_d_*S*_ test informs selection on a deeper timescale (Bamshad and Wooding 2003; Nielsen 2005; Mugal et al. 2014), the Ewens–Watterson test is especially suited for detecting recent selection events by comparing the observed homozygosity (*F*_obs_) with the expected homozygosity (*F*_exp_) (Nielsen 2001). Our Ewens–Watterson test results corroborate the possibility of selection limiting the diversity in centromeric *KIR* genes in Amerindians. The homozygosity was pronounced (*F*_obs_ > *F*_exp_) in several centromeric *KIR* genes in Amerindians, and a strikingly different pattern (*P *=* *4 × 10^−6^) was observed in the neighboring telomeric region (fig. 6*B*). In the Aché, significantly increased homozygosity was observed in five of the six centromeric genes (*P *>* *0.975). The Ewens–Watterson patterns corroborate the d_*N*__–_d_*S*_ deviations and suggest selective pressures on the centromeric *KIR* portion but not on the telomeric.

To further our analysis and eliminate the confounder effects of stochastic and demographic factors, we retrieved data from 678 genomic microsatellites (Msat) markers previously described in the Aché, Guarani, and Kaingang (Wang et al. 2007). We analyzed genome-wide diversity and population differentiation and compared them with those found in *KIR*. We observed that the overall differentiation in the Msat markers (mean *F*_ST_ = 0.0873) among these populations is higher than in *KIR* (fig. 6*C*). These significant differences (1.6 × 10^−4^ < *P *<* *1.1 × 10^−7^) would not be expected if the *KIR* region was under neutral evolution. Next, we analyzed the heterozygosity rates across the centromeric and telomeric *KIR* regions, comparing them with the heterozygosity of the genomic Msat. We found that the *KIR* centromeric heterozygosity is much lower than the genomic Msat heterozygosity in Amerindians (*P *=* *7 × 10^−4^), whereas the telomeric *KIR* region exhibits heterozygosity rates similar to the neutral genomic markers (fig. 6*D*). These results show a significantly different heterozygosity pattern between the centromeric and telomeric regions (*P *=* *4.7 × 10^−4^). Altogether, our observations indicate deviations from neutrality that cannot be fully explained by stochastic factors, pointing to a strong stabilizing selection specifically on the centromeric *KIR* region.

## Discussion

The uniqueness of the South American Amerindians is due to their singular demographic history of migration from Asian ancestral populations via the Behring Strait (Skoglund et al. 2015), followed by a complex dispersion along the American continent (Reich et al. 2012; Castro e Silva et al. 2020), as well as remaining genetically isolated during the last five centuries (Petzl-Erler et al. 1993; Tsuneto et al. 2003). The Amerindian demographic history is especially interesting because of the intense bottleneck and founder effects (Amos and Hoffman 2010; O’Fallon and Fehren-Schmitz 2011) and other stochastic effects intensified by their mostly small population sizes (Luiselli et al. 2000; Tarazona-Santos et al. 2001). Together, these events contributed to the low genomic diversity currently observed in Amerindian populations (DeGiorgio et al. 2009). Moreover, the unique genetic diversity of Amerindians may partially have emerged after the ancestral migration. A remarkable example is the episodic evolution in Amerindians from South America that replaced a large part of the ancestral *HLA* alleles with novel sets of population-specific *HLA* alleles never observed elsewhere (Belich et al. 1992; Watkins et al. 1992; Parham et al. 1997). Although describing such unique isolated populations is highly relevant, the genetic characterization of underrepresented admixed populations in South America is equally essential for anthropological genetics. Studies such as the one we present here lay the foundation for understanding the normal and pathologic human genetic variation and may contribute to creating personalized medicine solutions applicable to populations typically neglected in large genetic studies.

We found a limited number of *KIR* alleles in the Amerindians compared with the two urban populations. Aiming to compare the *KIR* diversity in Amerindians with global populations and due to the scarcity of high-resolution data available, we compared the study population with worldwide populations at three-digit resolution with allelic frequencies greater than or equal to 1%. We did not include rare alleles (*f *<* *1%) to consider only the most representative alleles of each population and reduce the bias of unbalanced sample sizes. We have shown a strong correlation of allele richness, a diversity measure that explicitly accounts for different sample sizes (Hurlbert 1971; el Mousadik and Petit 1996), with the number of common alleles. This correlation indicates that the number of three-digit *KIR* alleles with frequencies greater or equal to 1% is predictive of *KIR* allele richness in populations with unbalanced sample sizes.

Previously, the Yucpa from Colombia were regarded as exhibiting the lowest *KIR* diversity, with 25 *KIR* common alleles (Gendzekhadze et al. 2009). We observed similarly low diversity in the Brazilian Amerindians, with Kaingang of Rio das Cobras exhibiting an even smaller number of *KIR* alleles at three-digit resolution (*n* = 24). Looking specifically to the allele richness, Aché was the lowest diverse population (26.3), followed by Yucpa (28.9). In sharp contrast, the highest *KIR* diversity to date was observed in two African KhoeSan populations, Nama and Khomani, with 100 and 98 *KIR* alleles, respectively, and allele richness 78.3 and 78.9, respectively (Nemat-Gorgani et al. 2018). In the European-descent sample from our study, we found the *KIR* diversity was comparable with the observed in European descendants from the United States (Vierra-Green et al. 2012; Amorim et al. 2021). Our study is also the first to describe the allelic diversity of all *KIR* genes in individuals of Japanese ancestry. Our data allow us to conclude that the multiple Amerindian populations analyzed so far are those with the lowest *KIR* diversity, in agreement with previous studies limited to *KIR* gene-content level (Gendzekhadze et al. 2006; Augusto et al. 2013, 2015).

Nevertheless, we found 21 alleles in our Amerindian samples that were not present in the two urban Brazilian populations of European and Japanese ancestries. In the KIV alone, we observed six alleles not found in the other study populations. Four alleles not found in the two urban populations form the centromeric haplotype KIR3DL3*01406∼KIR2DL2*00602∼KIR2DL5B*00601∼KIR2DS5*004. This haplotype was previously described in the Ga-Adangbe from Ghana (Norman et al. 2013) and the KhoeSan from Southern Africa (Nemat-Gorgani et al. 2018). Similarly, the GKW presented three alleles that form the haplotype KIR2DL4*022∼KIR3DL1*024N∼KIR2DS4*00104, reported in the Ga-Adangbe from Ghana (Norman et al. 2007) in addition to KIR2DS4*00104, which was reported in African Americans (Hou et al. 2009). Interestingly, the allele KIR2DL5A*01201, first described in one African American individual (Hou et al. 2009), was found in all the study Amerindians (except the Aché), exhibiting frequencies from 0.6% to 4.2%. Our data suggest that some of the current alleles in the Amerindian populations may result from gene flow from African populations centuries ago. In fact, genomic-wide analysis indicates that the beginning of the admixture of Amerindians with Europeans and Africans started several generations ago when Brazil was still a Portuguese colony (Kehdy et al. 2015; Castro e Silva et al. 2020). However, our population differentiation and PCAs corroborate previous studies that show that despite a low level of gene flow, Amerindians from this study remained genetically isolated due to strong cultural barriers (Petzl-Erler et al. 1993; Tsuneto et al. 2003).

Amerindian populations did not group in the PCA, consistent with the intense demographic effects they experienced (Amos and Hoffman 2010; O’Fallon and Fehren-Schmitz 2011). For example, the three Guarani groups included in this study diverged despite sharing a common ancestor (Marrero et al. 2007); the differentiation among Guarani groups has also been seen for *HLA* and other immune markers (Tsuneto et al. 2003; Calonga-Solís et al. 2019). Therefore, the *KIR* differentiation observed in Amerindians is consistent with their history of intense genetic drift and is even more pronounced in Yucpa and Aché.

The description of high-resolution (five-digit) *KIR* haplotypes for all functional *KIR* genes and pairwise LD analysis were only achieved by one study that analyzed Euro-Americans (Amorim et al. 2021). We observed population-specific patterns of allelic pairwise LD, which highlights the uniqueness of the study populations. Our current study contributes to revealing the still unknown LD patterns of *KIR* alleles in global populations. We found more variation in copy number arrangements, including deletions, duplications, and hybrid gene formation in telomeric haplotypes than we found in centromeric haplotypes. This observation is consistent with previous suggestions that there is a greater selective advantage in the diversification of gene content (presence and absence of genes) and large structural variations in the telomeric region (Jiang et al. 2012). Among the uncommon haplotypes, we observed deletion of the framework gene *KIR3DL2* in the haplotype *cA03∼tB07* in Euro-Brazilians and Japanese descendants (f = 1.38 and 0.69%, respectively), along with the deletion of *KIR2DL1∼KIR3DP1∼KIR2DL4∼KIR3DS1∼KIR2DL5∼KIR2DS35.* This pattern possibly represents a fusion of *KIR2DL1* and *KIR2DS1* with the deletion of the framework gene *KIR3DL2* (Pyo et al. 2013).

In contrast, we found a remarkably low diversity in the Amerindian centromeric *KIR* haplotypes. For example, in Aché, the gene-content haplotype *cA01* was observed in 100% of the individuals, with a minor allelic variation. Interestingly, a study analyzing single-nucleotide polymorphisms within genes of the innate immune system (Lindenau et al. 2016) reported reduced heterozygosity in Aché regardless of the similar genomic microsatellites heterozygosity compared with Guarani and Kaingang (Wang et al. 2007).

Because the centromeric *KIR* region encodes the strongest educators for NK cells (Stewart et al. 2005; Hilton et al. 2015), we hypothesize that the reduced diversity in this region is a consequence of selective pressure imposed by the limited *HLA* alleles in these populations. We and others have already reported frequencies of the KIR ligands in Brazilians of European ancestry (Augusto et al. 2012), Japanese ancestry (Augusto et al. 2016), and some Brazilian Amerindians (Parham et al. 1997; Augusto et al. 2013), while we have characterized Aché here for the first time. We observed a limited number of KIR–HLA interactions per individual in the Amerindians from our study, particularly the GRC, which has the lowest score ever reported for a human population, 2.86. Most striking is the scarcity of HLA-A and HLA-B ligands, which causes 85–97% of all KIR–HLA interactions in all studied Amerindians to rely almost exclusively on HLA-C. These results support the suggestion that HLA-C specialized and evolved to become primarily KIR ligands whereas HLA-A and HLA-B kept their primary function as T-cell receptor ligands (Older Aguilar et al. 2010; Augusto et al. 2015).

Gendzekhadze et al. (2009) earlier suggested that the limited diversity of KIR–HLA in Amerindians may be the minimum necessary for human survival. We further hypothesize that the reduced KIR–HLA interactions might have selected specific sets of *KIR* centromeric variants in these populations. We observed an excess of synonymous to nonsynonymous substitutions in the centromeric region and significantly increased homozygosity in many centromeric loci, both indicating purifying selection. Conversely, this was not observed in the telomeric portion, and the differences in synonymous and nonsynonymous nucleotide substitution rates in the telomeric region were similar to the observed in the majority of other protein-coding genes (Kryazhimskiy and Plotkin 2008; Dasmeh et al. 2014).Although significant, both d_*N*__–_d_*S*_ and Ewens–Watterson analyses may not alone overrule the occurrence of genetic drift and other stochastic factors. To neutralize these confounding variables, we compared the differentiation and homozygosity of the *KIR* region with 678 neutral genomic markers (Wang et al. 2007). The observation that Amerindians are less differentiated for *KIR* than for the neutral genomic markers is an additional signature of selection. Lastly, we found that the homozygosity in the centromeric *KIR* region is much lower than the homozygosity in the telomeric region compared with genomic homozygosity rates. Once again, these last two observations are robust signatures of purifying selection in centromeric *KIR* but not in telomeric *KIR*. Our observations corroborate previous findings showing a geographically specific selection on the *KIR* complex, particularly on the centromeric region (Yawata et al. 2006; Augusto et al. 2019; Deng et al. 2021). Thus, we suggest that the limited number of HLA–KIR interactions, represented mainly by few HLA-C ligands, impose an intense population-specific stabilizing pressure on the *KIR* centromeric region to maintain the minimal inhibitory signals required for NK education and the consequent survival of these populations. Population-specific selection of *KIR*–*HLA* combinations driven by distinct sets of *HLA* alleles could also have contributed to the greater differentiation observed for the centromeric *KIR* in our Amerindians compared with the telomeric.

In conclusion, we provide a comprehensive and novel characterization of *KIR* at high resolution in unique populations, also in the context of their HLA ligands. For the first time, we report high-resolution allele-level haplotypes in South American populations, including six Amerindian and two Southern Brazilian urban populations. Importantly, we provide compelling evidence of purifying selection on *KIR* centromeric haplotypes, possibly driven by the reduced diversity of *HLA* alleles. Genetic characterization of such unique populations is of paramount interest to understand the evolutionary constraints and impacts of reduced diversity in human populations. This study significantly contributes to understanding high-resolution allelic and haplotypic *KIR* variation in global populations, bringing new insights into LD patterns among *KIR* alleles and enhancing our ability to identify *KIR* haplotypes.

## Materials and Methods

### Characterization of the Study Populations

**Fig. 7.**
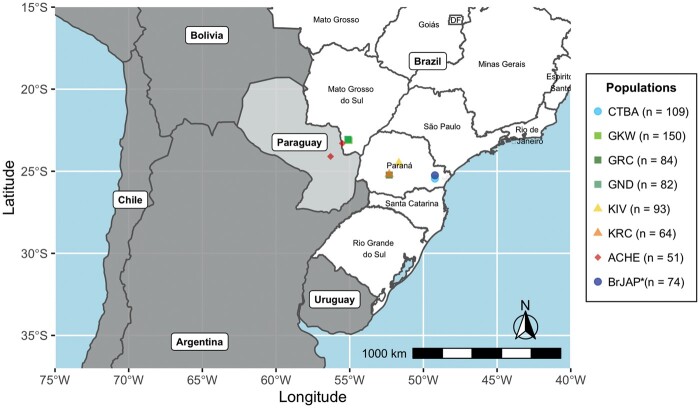
Characterization of the study populations. Map of the geographic locations of the eight populations included in this study. The territory in white corresponds to Brazil, light gray highlights Paraguay, and other countries are indicated in dark gray. Sample sizes are shown inside parenthesis. The study populations include Brazilians of European ancestry (CTBA); Brazilians of Japanese ancestry (BrJAP); Aché (ACHE); GKW; GND; GRC; KIV; and KRC. *The BrJAP population is comprised of individuals whose all ancestors were born in Japan, without known admixture with non-Japanese. For detailed information, see supplementary table 1, Supplementary Material online.

All participants were informed about the research purpose and given written or oral consent to participate in the study, according to the local law and regulation at the time of sample collection. This study was approved by the Human Research Ethics Committee of the Federal University of Paraná and the Brazilian National Human Research Ethics Committee (CONEP), protocol number CAAE 02727412.4.0000.0096, under the Brazilian Federal laws. We analyzed 706 individuals from eight different populations (fig. 7). They include Euro-descendants from Curitiba (CTBA, *n* = 109), Japanese descendants from Curitiba (BrJAP, *n* = 74), GKW (*n* = 150), GND (*n* = 81), GRC (*n* = 84), KIV (*n* = 93), KRC (*n* = 64), and the Aché (ACHE, *n* = 51). As described previously, Amerindian sample collection occurred between the late 1980s and early 1990s (Tsuneto et al. 2003). Detailed information of the study populations is given in supplementary table 11, Supplementary Material online, and figure 7.

All Amerindian individuals were part of genetically isolated groups living in indigenous lands in the Brazilian states of Paraná, and Mato Grosso do Sul, and in the bordering country Paraguay. The Guarani populations are closely related groups and speak dialects of a common language, Guarani, from the Tupi-Guarani linguistic family. They are subdivided into GND, GKW, and GRC, which diverged around 1,800 years ago. In contrast, the Kaingang populations speak a language belonging to the Jê family and were suggested to have split only approximately 200 years ago (Marrero et al. 2007). The Aché, also known as Guayaki, live in Paraguay and also speak a language belonging to the Tupi-Guarani family, sharing some cultural similarities with Guarani and Kaingang groups. However, autosomal and sexual genetic markers suggest they are closer to Guarani groups than Kaingang (Battilana et al. 2002; Gaspar et al. 2002; Tsuneto et al. 2003; Schmitt et al. 2004; Callegari-Jacques et al. 2007).

The urban populations were collected in Curitiba, Paraná State, one of Brazil's largest cities, with over 2 million inhabitants. Southern Brazil was initially inhabited by Amerindians and later by Africans brought enslaved by the Portuguese during the colonization period. Later, this region received a large influx of European immigrants during the XIX and XX centuries (Santos 2002; Kehdy et al. 2015; Pena et al. 2020). The population of Curitiba has predominantly European ancestry, with mainly Portuguese, German, Italian, Polish, and Ukrainian backgrounds, among others. According to the Brazilian population census in 2010 (IBGE 2013), 78.8% of Curitiba's population self-declared as Euro-descendants, 16.7% admixed, 3% Afro-descendants, 1.4% Asian, and 0.2% Native American.

During Japan's crisis after the Meiji Restoration in the late XIX century, there was a large immigration of Japanese people to Brazil, the United States, Peru, and Mexico (Sakurai et al. 2010). Currently, Brazil hosts the largest Japanese population outside Japan, with over 1.5 million Japanese descendants living mainly in São Paulo and Paraná States (IBGE 2013). Paraná hosts the second-largest Japanese community in Brazil, comprised of over 30,000 Japanese or Japanese descendants, which remain relatively isolated. All Japanese-descendant individuals included in this study were born in Curitiba, Paraná, and reported that all four grandparents were born in Japan without known admixture with non-Japanese ancestries.

### *KIR* and *HLA* Sequencing

DNA was extracted using the phenol-chloroform-isoamyl alcohol method (Sambrook et al. 1989) or the salting-out method (Lahiri and Nurnberger 1991) and stored at −80 °C. DNA was enzymatically fragmented using the KAPA HyperPlus kit (Roche, USA) and barcoded with unique adaptors. Dual size selection was performed with AMPure XP magnetic beads (Beckman Coulter, USA) to obtain fragments with an average size of 780 bp. Quality control was performed using the PicoGreen kit (Thermo Fisher Scientific, Waltham, MA) for product quantification and Bioanalyzer (Agilent, Santa Clara, CA) to determine the quantity and size of fragments. Pooling was performed with the automated liquid handler Echo 525 (Labcyte, San Jose, CA). The enrichment of the targeted regions was performed with the Nextera kit (Illumina, San Diego, CA) using 10,456 biotinylated probes designed by Norman et al. (2016) to capture fragments corresponding to all *KIR* and *HLA* class I loci. After this step, the fragments were purified and amplified. Sequencing was performed using Illumina HiSeq 4000 (Illumina, San Diego, CA) 150 bp paired-end protocol.

### Data Analysis

Sequence filtering, alignment, and genotype calling of *KIR* genes were made using an updated version of the PING bioinformatic pipeline (Norman et al. 2016; Marin et al. 2021). Genotype and copy number were obtained for all *KIR* loci, except the two pseudogenes for which we only determined copy number. After processing with PING, *KIR* data were manually curated for the resolution of ambiguities. For *HLA-A*, *HLA-B*, and *HLA-C* genotyping, we processed raw FASTQ files with HLA Explore (Omixon, Hungary), which determined unambiguous calls for three-field resolution genotypes.

Allele frequencies, allelic richness, and the proportion of shared *KIR* alleles among populations were calculated and plotted using a custom version of the *PopGenReport* R package (Adamack and Gruber 2014). Intersecting sets of alleles were identified using *intersect* from base R and plotted using *upset* from R package UpSetR (Conway et al. 2017). LD between the multiallelic *KIR* loci was performed on Arlequin version 3.5.2 (Excoffier and Lischer 2010). Population pairwise *F*_ST_ was calculated with *PopGenReport* and locus-specific *F*_ST_ with *pegas* R packages (Weir and Cockerham 1984; Paradis 2010; Adamack and Gruber 2014). The neighbor-joining tree was estimated in the R package *ape* (Paradis and Schliep 2019). The significance of the genetic differentiation between populations was tested using the exact test of population differentiation (Raymond and Rousset 1995; Goudet et al. 1996) on Arlequin version 3.5.2 (Excoffier and Lischer 2010). Principal components analysis was performed and plotted using the *ade4* R package (Dray and Dufour 2007). Maps were plotted using the R packages *maps* (Becker et al. 2018) and *geobr* (Pereira and Gonçalves 2020). Neutrality tests of nonsynonymous (d_*N*_) to synonymous (d_*S*_) substitutions (Nei and Gojobori 1986) were performed in MEGA X software (Kumar et al. 2018). The statistical significance of the difference was tested using the bootstrap method with 1,000 replicates and performing the two-tailed Z-test of selection, in which Z = (d_*N*__–_d_S_)/SQRT(Var(d_*S*_) + Var(d_*N*_)) (Kumar et al. 2018). The Ewens–Watterson test was performed with 1,000 replicates on Arlequin version 3.5.2 (Excoffier and Lischer 2010), with Slatkin’s probability correction for multiallelic data (Ewens 1972; Watterson 1978; Slatkin 1994; 1996). For this test, significant deviation toward homozygosity is indicated by probability values close to one, and heterozygote excess is indicated with values close to zero. Therefore, we established the significant thresholds as *P *<* *0.025 or *P *>* *0.975, considering a cumulative error of 5% for the Ewens–Watterson test. Heterozygosity values were obtained using R package *adegenet* (Jombart 2008).

We defined *KIR* centromeric and telomeric haplotypes at the gene-content level according to the nomenclature described previously (Pyo et al. 2010; Vierra-Green et al. 2012). Furthermore, we included the nomenclature proposed in Pyo et al. (2013) to describe unusual haplotype patterns (e.g., *tB01-del6*, which represents *KIR3DP1-KIR2DL4-KIR3DS1* deletion). The identification of centromeric and telomeric haplotypes, including the allele-level *KIR* haplotype determination, were performed manually based on known patterns (Vierra-Green et al. 2012; Pyo et al. 2013; Roe et al. 2017).

*HLA-A*, *HLA-B*, and *HLA-C* alleles were classified according to their encoded relevant epitopes for KIR interaction. *HLA* and *KIR* data were integrated to generate individual interaction scores described previously (Nemat-Gorgani et al. 2018). We considered the following pairs: Bw4 (HLA-A) and KIR3DL1 (Foley et al. 2008); Bw4 (HLA-B) and KIR3DL1 (Gumperz et al. 1995; Foley et al. 2008); HLA-A*03 and KIR3DL2 (Döhring et al. 1996); HLA-A*11 and KIR3DL2 (Hansasuta et al. 2004); HLA-C2 e KIR2DL1 (Hilton et al. 2015); HLA-C1 and KIR2DL2 (Hilton et al. 2015); HLA-C1 and KIR2DL3 (Hilton et al. 2015); HLA-C2 and KIR2DS1 (Hilton et al. 2015); HLA-C*16 and KIR2DS2 (Moesta et al. 2010); HLA-A*11 and KIR2DS4 (Graef et al. 2009); a subset of HLA-C which includes C2 (alleles HLA-C*05:01, *02:02, and *04:01) and C1 (alleles HLA-C*16:01, *01:02, and *14:02), recognized by KIR2DS4 (Graef et al. 2009); and, finally, HLA-C2 and a subset of KIR2DS5 receptors (encoded by alleles KIR2DS5*003, *004, *005, *006, *007, and *008) (Blokhuis et al. 2017).

## Supplementary Material

Supplementary data are available at *Molecular Biology and Evolution* online.

## Supplementary Material

msab298_Supplementary_Data
