# Supplementary material for: Remarkably Low KIR and HLA Diversity in Amerindians Reveals Signatures of Strong Purifying Selection Shaping the Centromeric KIR Region
Source: Mol Biol Evol. 2021 Oct 11;39(1):msab298. doi: 10.1093/molbev/msab298 (PMC8763117; doi:10.1093/molbev/msab298)
Supplement: msab298_Supplementary_Data [file msab298_supplementary_data.zip › 2021_Vargas_Supplementary_rev.pdf]

Supplementary Information

Remarkably low *KIR* and *HLA* diversity in Amerindians reveals signatures of strong purifying selection shaping the centromeric *KIR* region.

Luciana de Brito Vargas[1], Márcia H. Beltrame[1], Brenda Ho[2], Wesley M. Marin[2], Ravi Dandekar[2], Gonzalo Montero-Martín[3], Marcelo A. Fernández-Viña[3], A. Magdalena Hurtado[4], Kim R. Hill[4], Luiza T. Tsuneto[5], Mara H. Hutz[6], Francisco M. Salzano[6,†], Maria Luiza Petzl-Erler[1], Jill A. Hollenbach[2,7], Danillo G. Augusto[1,2*]

[1]Programa de Pós-Graduação em Genética, Departamento de Genética, Universidade Federal do Paraná, Curitiba, PR 81531-980, Brazil; [2]Weill Institute for Neurosciences, Department of Neurology, University of California, San Francisco, San Francisco, CA 94158, USA; [3]Department of Pathology, Stanford University School of Medicine, Palo Alto, CA 94304, USA; [4]School of Human Evolution and Social Change, Arizona State University, Tempe, AZ 85287, USA; [5]Departamento de Análises Clínicas, Universidade Estadual de Maringá, Maringá, PR 87020-900, Brazil; [6]Departamento de Genética, Instituto de Biociências, Universidade Federal do Rio Grande do Sul, Porto Alegre, RS 91501-970, Brazil. [7]Department of Epidemiology and Biostatistics, University of California, San Francisco.

† Deceased September 28, 2018.

***Corresponding author:** Danillo G. Augusto
E-mail: danillo@augusto.bio.br

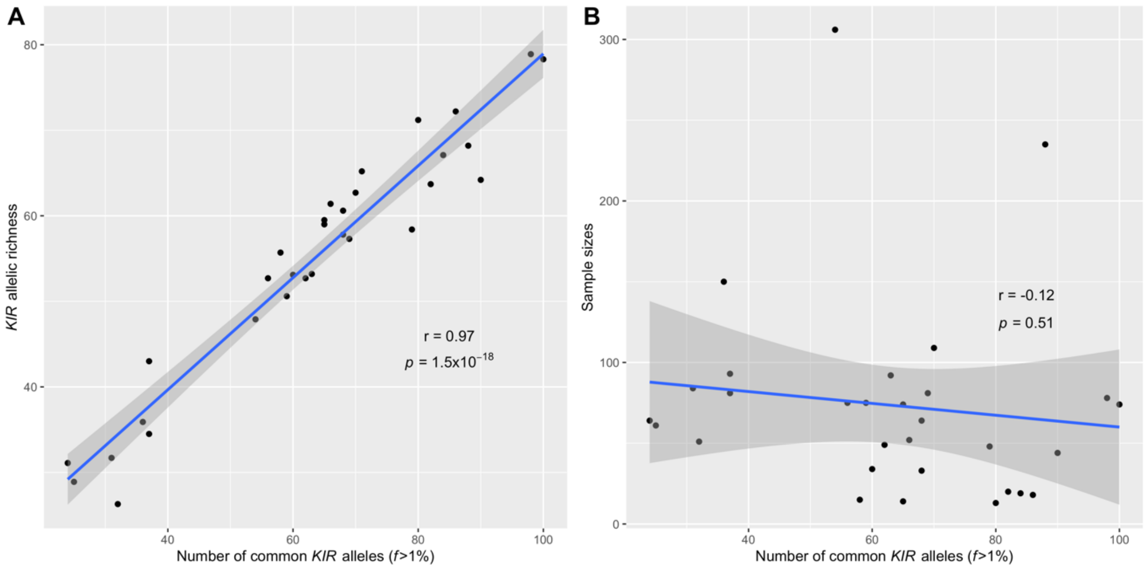

Supplementary Figure 1. Number of common alleles is predictive of allele richness for *KIR*. **A)** Number of *KIR* alleles with frequencies equal or greater than 1% strongly correlates with allele richness in global populations. Each dot represents a population studied for *KIR*, further detailed in Supplementary Table S4 and Figure 2. **B)** Number of common *KIR* alleles, with frequencies equal or greater than 1%, does not correlate with the population sample sizes. Each dot represents a population from Supplementary Table 4 and Figure 2.

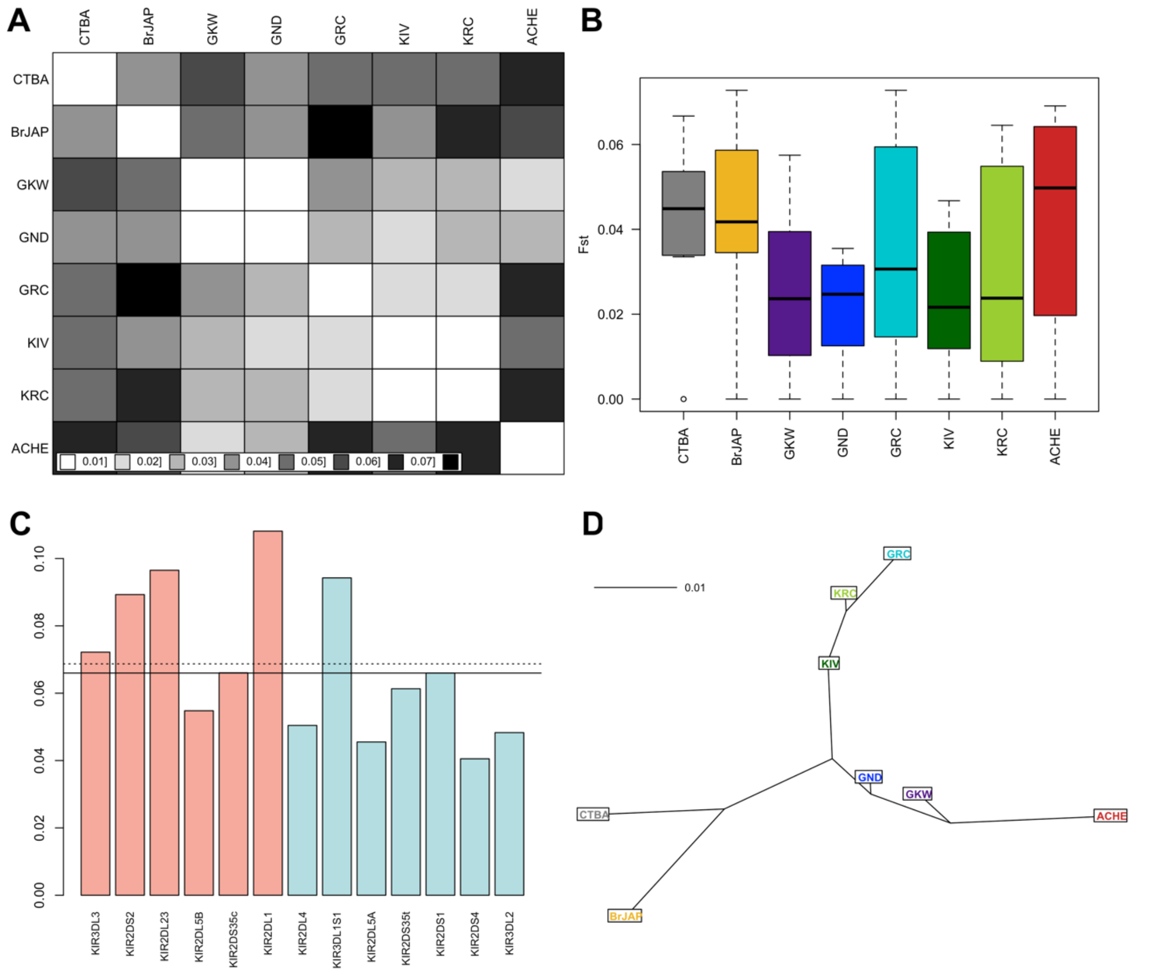

Supplementary Figure 2. Population differentiation of the *KIR* allelic variation. A) Gray scale table of pairwise F_{ST} differentiation values. Values can indicate low (<0.05), medium (0.05 to 0.15) or high (>0.15) differentiation between populations (2). **B)** Box plot showing the distribution of differentiation values found for each population among the pairwise F_{ST} values. The line marks the median F_{ST} value, and the upper and bottom limits of the box mark the superior and inferior quartiles, respectively. Whiskers represent minimum and maximum F_{ST} values found. **C)** Average F_{ST} differentiation in each *KIR locus*. The continuous line indicates the average F_{ST} across all populations and the dotted line indicates the median F_{ST}. Light red: centromeric *KIR* genes. Light blue: telomeric *KIR* genes. **D)** Genetic distance tree of F_{ST} values, estimated with the neighbor-joining (NJ) method. CTBA: Brazilians of European ancestry. BrJAP: Brazilians of Japanese ancestry. ACHE: Aché. GKW: Guarani Kaiowá. GND: Guarani Ñandeva. GRC: Guarani Mbya. KIV: Kaingang from Ivaí. KRC: Kaingang from Rio das Cobras.

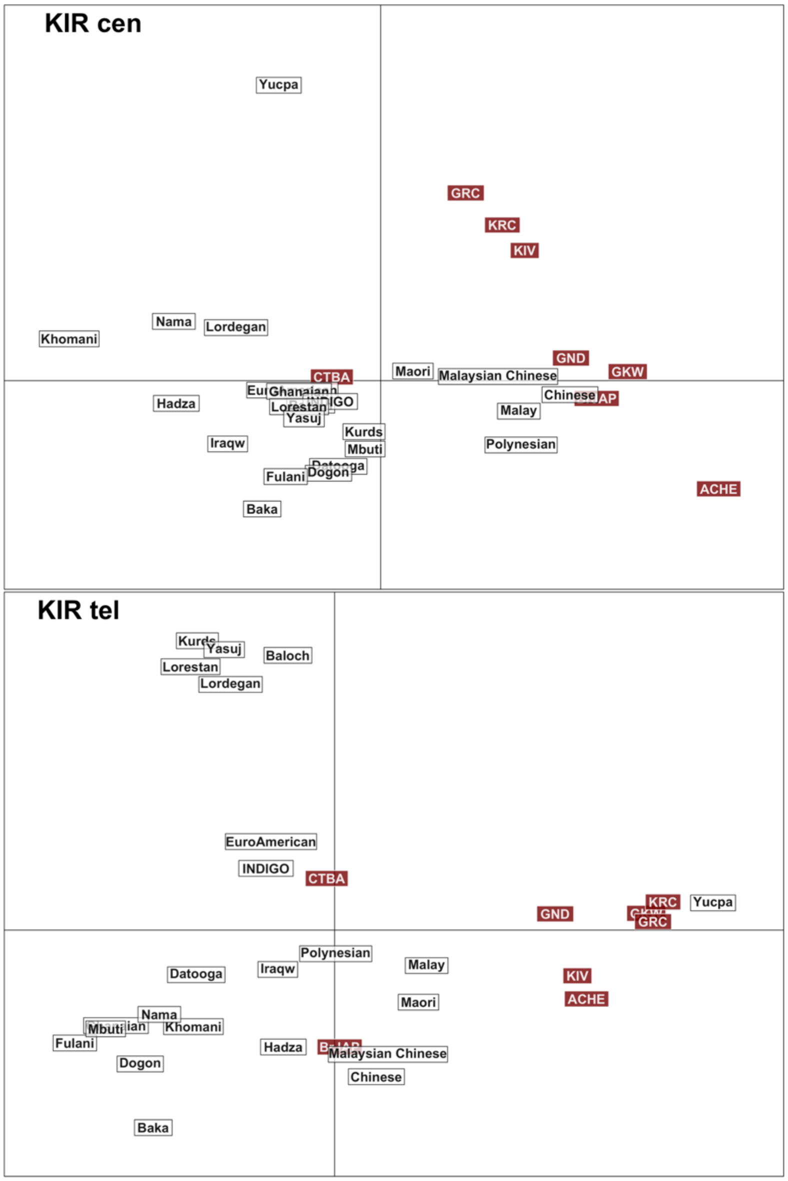

Supplementary Figure S3. Principal components analysis (PCA) of *KIR* variation. Here, PCA analysis was run separately for centromeric *KIR* (KIR cen, top) and telomeric KIR (KIR tel, bottom) genes. The PCA analysis included 31 world populations studied at the allele level for all *KIR* genes. To maximize our analysis, we included studies that described *KIR* variation at 3-digit allelic resolution. Curitiba (CTBA); Brazilians of Japanese ancestry (BrJAP); Aché (ACHE); Guarani Kaiowá (GKW); Guarani Ñandeva (GND); Guarani Mbya (GRC); Kaingang from Ivaí (KIV); and Kaingang from Rio das Cobras (KRC). Data from previously analyzed populations are found in (Gendzekhadze et al. 2009; Vierra-Green et al. 2012; Norman et al. 2013b; Nemat-Gorgani et al. 2014; Nemat-Gorgani et al. 2018; Nemat-Gorgani et al. 2019; Alicata et al. 2020; Tao et al. 2020; Amorim et al. 2021; Deng et al. 2021)

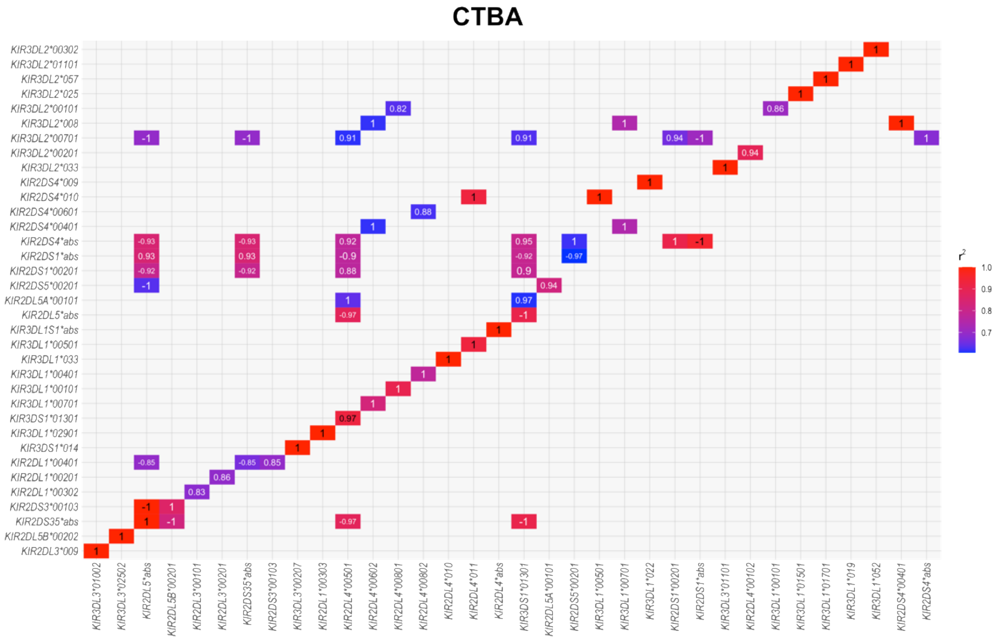

Supplementary Figure 4. Pairwise linkage disequilibrium (LD) between *KIR* alleles in European descendants from Curitiba (CTBA). Boxes represent the pairs of *KIR* alleles with the strongest LD in the present study. D' values are written inside of each box and color scale represent r^2 values. Only pairs with $r^2 > 0.6$, D' > |0.6| are shown. The *p*-value of all pairs was < 10^{-6}.

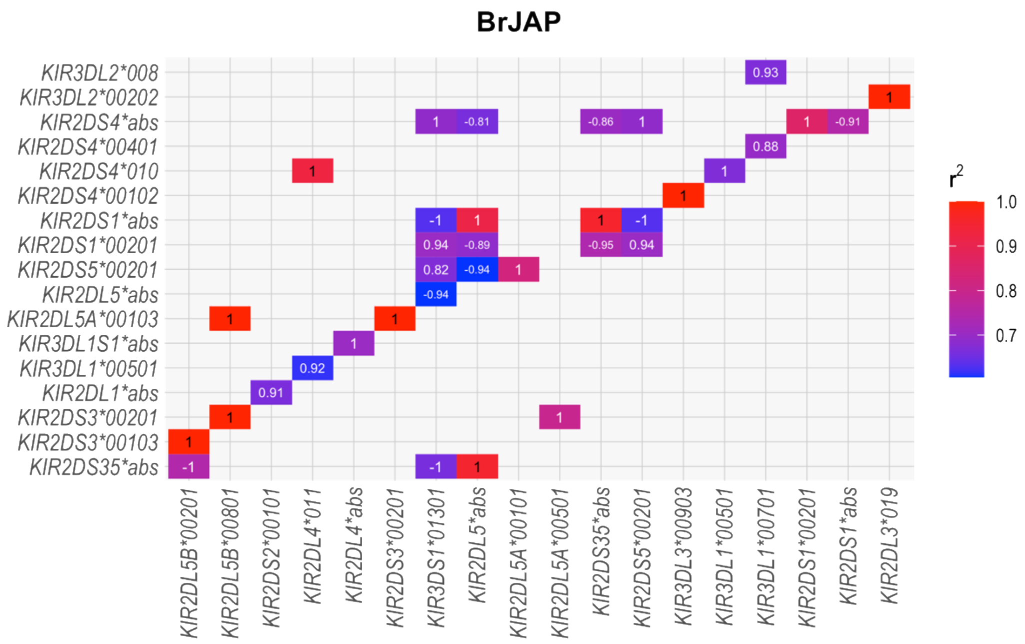

Supplementary Figure 5. Pairwise linkage disequilibrium (LD) between *KIR* alleles in Brazilian Japanese (BrJAP). Boxes represent the pairs of *KIR* alleles with the strongest LD in the present study. D' values are written inside of each box and color scale represent r^2 values. Only pairs with $r^2 > 0.6$, D' > |0.6| are shown. The *p*-value of all pairs was $< 10^{-6}$.

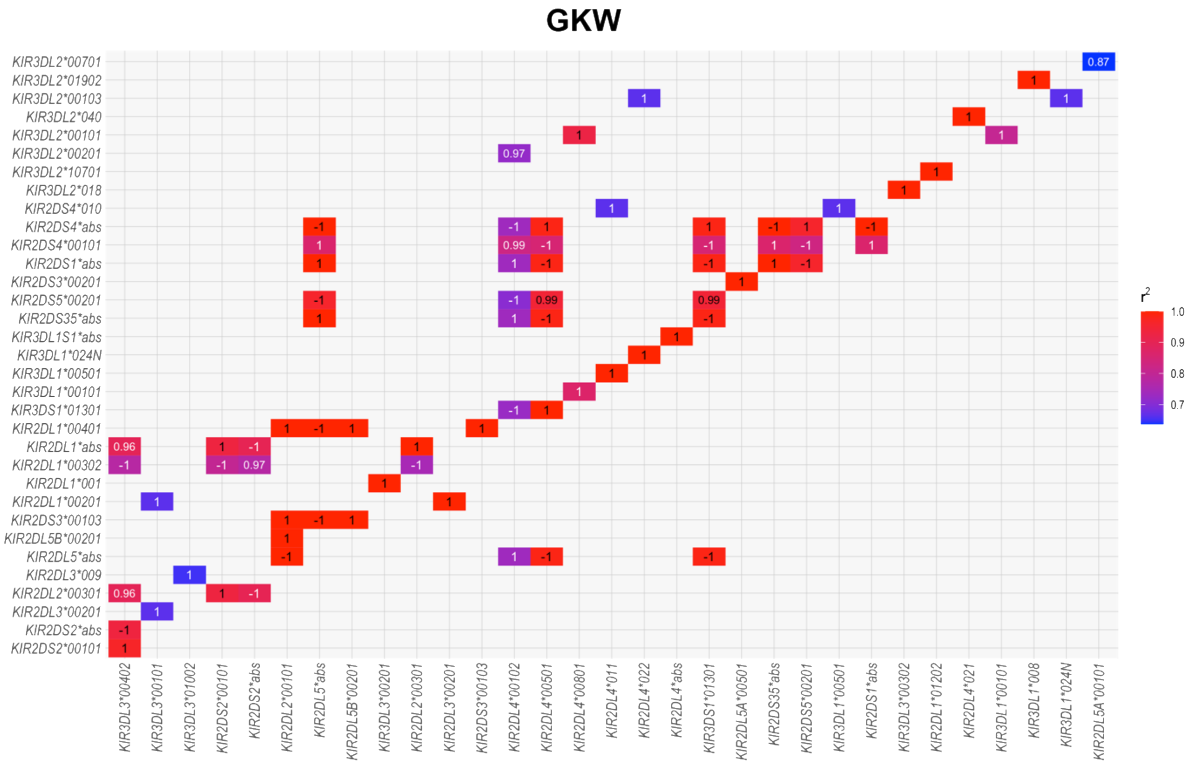

Supplementary Figure 6. Pairwise linkage disequilibrium (LD) between *KIR* alleles in Guarani Kaiowá (GKW). Boxes represent the pairs of *KIR* alleles with the strongest LD in the present study. D' values are written inside of each box and color scale represent r2 values. Only pairs with r2 > 0.6, D' > |0.6| are shown. The p-value of all pairs was < 10^{-6}.

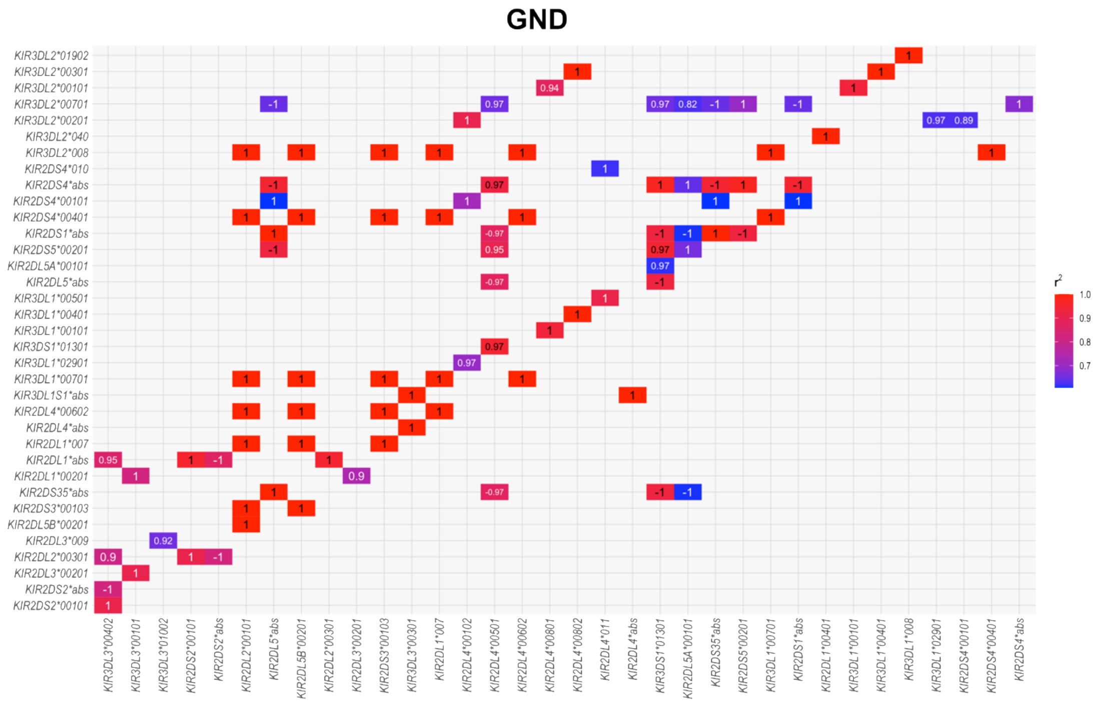

Supplementary Figure 7. Pairwise linkage disequilibrium (LD) between *KIR* alleles in Guarani Ñandeva (GND). Boxes represent the pairs of *KIR* alleles with the strongest LD in the present study. D’ values are written inside of each box and color scale represent r^2 values. Only pairs with $r^2 > 0.6$, D’ > |0.6| are shown. The *p*-value of all pairs was < 10^{-6}.

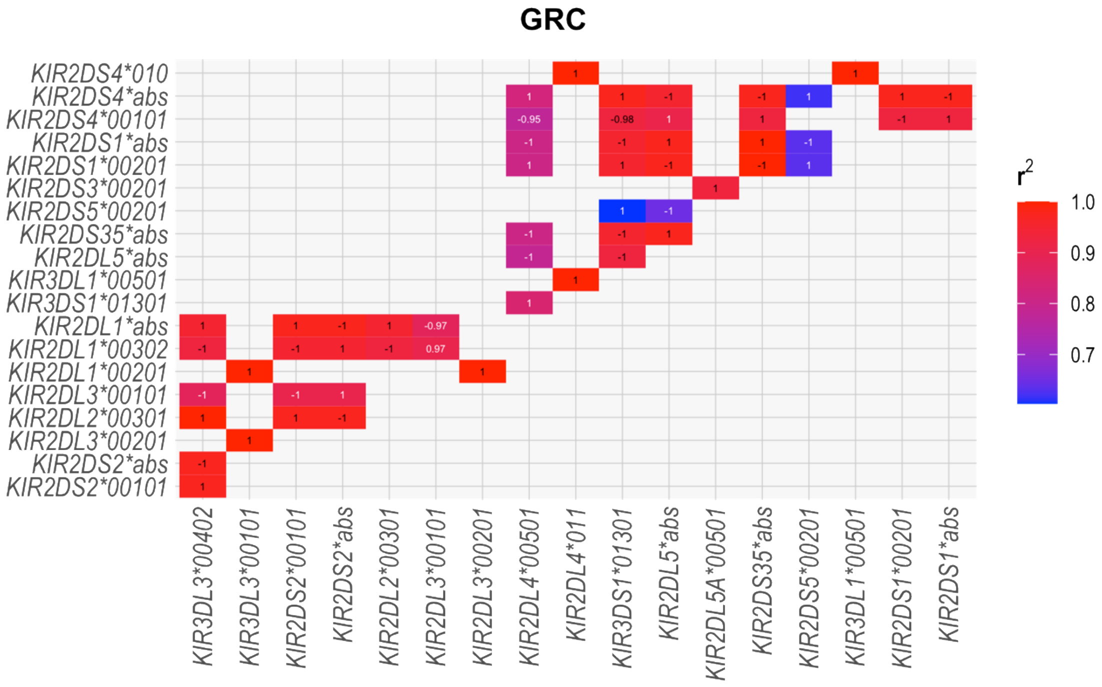

Supplementary Figure 8. Pairwise linkage disequilibrium (LD) between *KIR* alleles in Guarani Mbya (GRC). Boxes represent the pairs of *KIR* alleles with the strongest LD in the present study. D’ values are written inside of each box and color scale represent r^2 values. Only pairs with $r^2 > 0.6$, D’ > |0.6| are shown. The *p*-value of all pairs was $< 10^{-6}$.

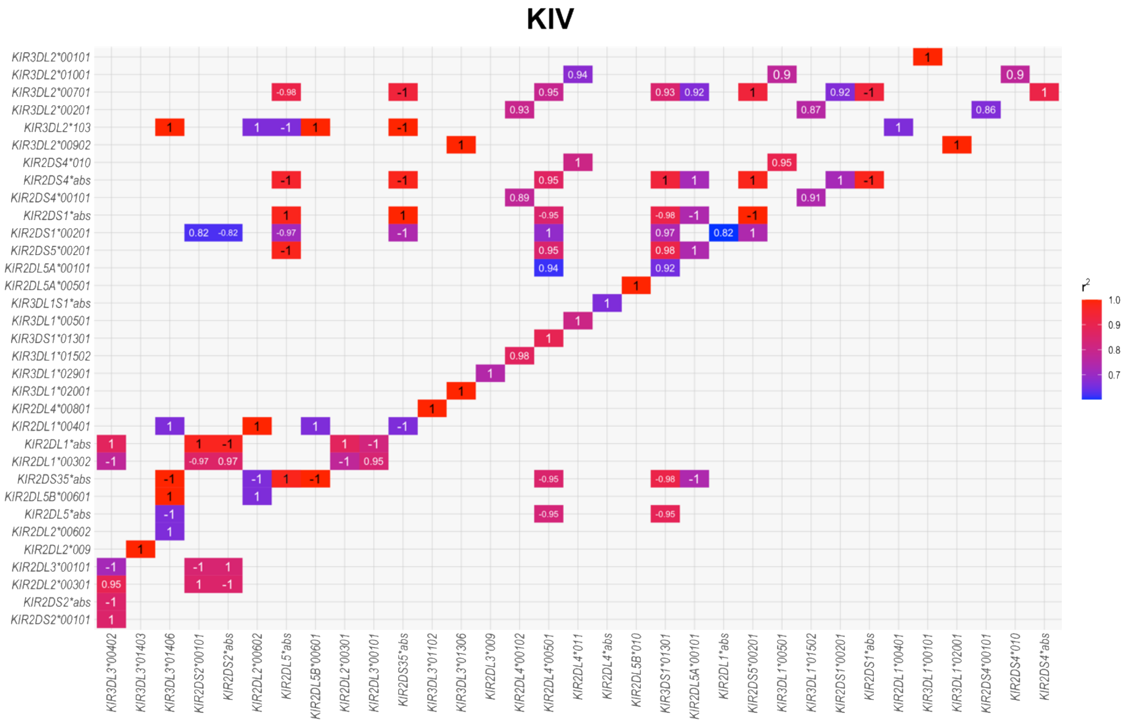

Supplementary Figure 9. Pairwise linkage disequilibrium (LD) between *KIR* alleles in Kaingang from Ivaí (KIV). Boxes represent the pairs of *KIR* alleles with the strongest LD in the present study. D' values are written inside of each box and color scale represent r^2 values. Only pairs with $r^2 > 0.6$, D' > |0.6| are shown. The *p*-value of all pairs was $< 10^{-6}$.

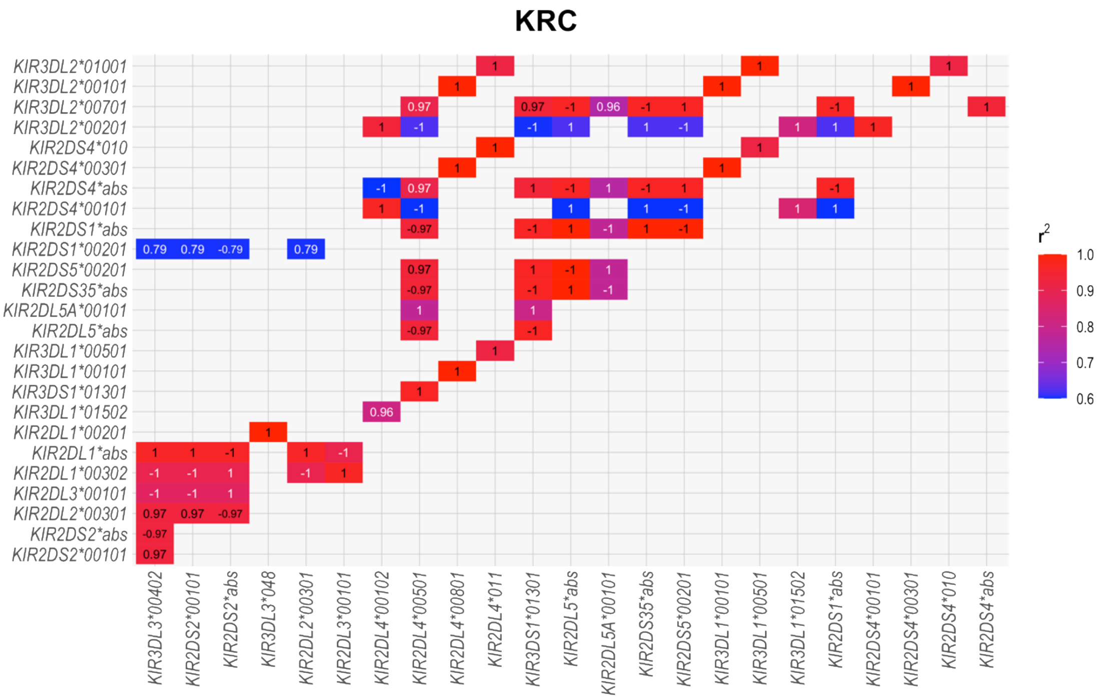

Supplementary Figure 10. Pairwise linkage disequilibrium (LD) between *KIR* alleles in Kaingang from Rio das Cobras (KRC). Boxes represent the pairs of *KIR* alleles with the strongest LD in the present study. D' values are written inside of each box and color scale represent r^2 values. Only pairs with $r^2 > 0.6$, D' > |0.6| are shown. The *p*-value of all pairs was < 10^{-6}.

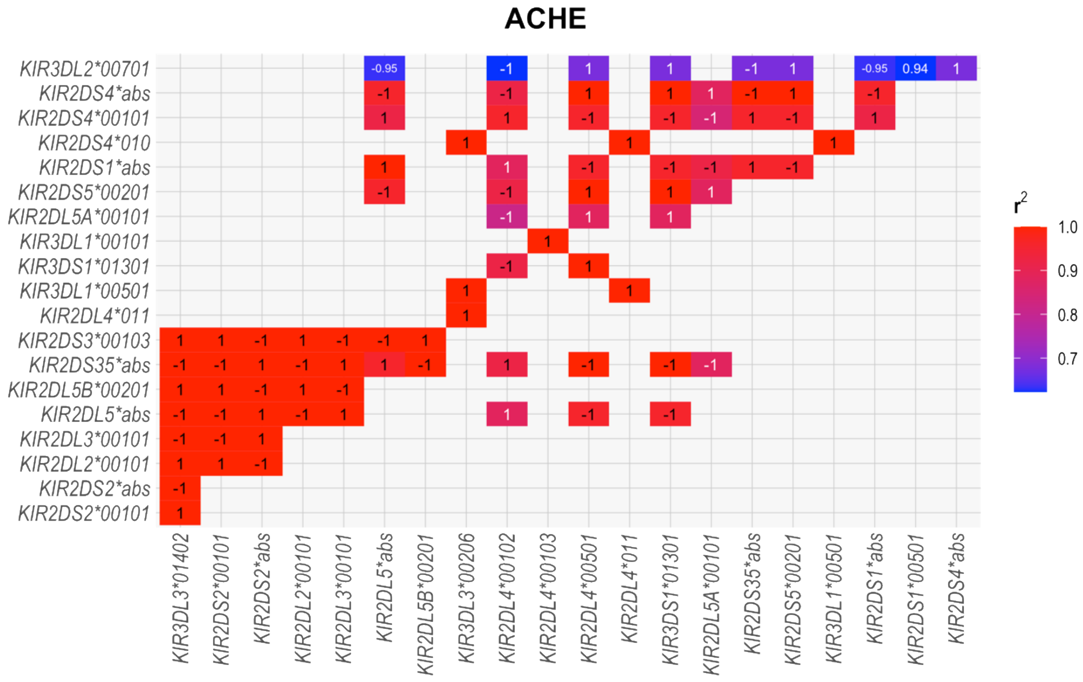

Supplementary Figure 11. Pairwise linkage disequilibrium (LD) between *KIR* alleles in Aché. Boxes represent the pairs of *KIR* alleles with the strongest LD in the present study. D' values are written inside of each box and color scale represent r^2 values. Only pairs with $r^2 > 0.6$, D' > |0.6| are shown. The *p*-value of all pairs was < 10^{-6}.

Dataset S1 (Excel file) with

Supplementary Table 1. Detailed frequencies of all 199 *KIR* variants in the study populations. The study populations include Brazilians of European ancestry (CTBA); Brazilians of Japanese ancestry (BrJAP); Aché (ACHE); Guarani Kaiowá (GKW); Guarani Ñandeva (GND); Guarani Mbya (GRC); Kaingang from Ivaí (KIV); and Kaingang from Rio das Cobras (KRC).

Supplementary Table 2. Alleles commonly found in the Amerindians, but absent or rare in the urban populations of European and Japanese ancestry. The study populations include Brazilians of European ancestry (CTBA); Brazilians of Japanese ancestry (BrJAP); Aché (ACHE); Guarani Kaiowá (GKW); Guarani Ñandeva (GND); Guarani Mbya (GRC); Kaingang from Ivaí (KIV); and Kaingang from Rio das Cobras (KRC).

Supplementary Table 3. Population differentiation in *KIR*. Lower diagonal: Proportion of shared *KIR* alleles among populations. These values are based on the minimum frequency of each allele between two populations, the minor frequencies for all alleles of a given locus are then summed. This sum is then averaged across all *KIR* loci to give the pairwise proportion of shared alleles. Upper diagonal: Statistical significance of the exact test of population differentiation between populations (Raymond and Rousset 1995; Goudet et al. 1996). * $p < 0.001$; ns (not significant) $p > 0.05$. CTBA: Brazilians of European ancestry. BrJAP: Brazilians of Japanese ancestry. ACHE: Aché. GKW: Guarani Kaiowá. GND: Guarani Ñandeva. GRC: Guarani Mbya. KIV: Kaingang from Ivaí. KRC: Kaingang from Rio das Cobras.

Supplementary Table 4. *KIR* allelic diversity in worldwide populations. The study populations are highlighted in red. # Numbers are relative to Fig 2. *KIR2DS35c, KIR2DS35t, KIR2DP1* and *KIR3DP1* loci were excluded from analyses because they are not described in all studies.

Supplementary Table 5. Allele-level centromeric *KIR* haplotypes (*KIR3DL3 ~ KIR2DL1*) found in South American populations. Haplotypes that were observed at least three times were included. On the right side of each haplotype is the *KIR* gene-content haplotype nomenclature as previously described (Pyo et al. 2010; Vierra-Green et al. 2012), and the frequency each haplotype was found in the populations. CTBA: Brazilians of European ancestry. BrJAP: Brazilians of Japanese ancestry. ACHE: Aché. GKW: Guarani Kaiowá. GND: Guarani Ñandeva. GRC: Guarani Mbya. KIV: Kaingang from Ivaí. KRC: Kaingang from Rio das Cobras.

Supplementary Table 6. Allele-level telomeric *KIR* haplotypes (*KIR2DL4 ~ KIR3DL2*) found in South American populations. Haplotypes that were observed at least three times were included. On the right side of each haplotype is the *KIR* gene-content haplotype nomenclature as previously described (Pyo et al. 2010; Vierra-Green et al. 2012), and the frequency each

haplotype was found in the populations. CTBA: Brazilians of European ancestry. BrJAP: Brazilians of Japanese ancestry. ACHE: Aché. GKW: Guarani Kaiowá. GND: Guarani Ñandeva. GRC: Guarani Mbya. KIV: Kaingang from Ivaí. KRC: Kaingang from Rio das Cobras.

Supplementary Table 6. Frequencies of gene-content *KIR* haplotypes found in the eight South American populations. Haplotypes that were observed at least three times were included. The nomenclature of *KIR* gene-content haplotypes used is as previously described (6, 7). CTBA: Brazilians of European ancestry. BrJAP: Brazilians of Japanese ancestry. ACHE: Aché. GKW: Guarani Kaiowá. GND: Guarani Ñandeva. GRC: Guarani Mbya. KIV: Kaingang from Ivaí. KRC: Kaingang from Rio das Cobras.

Supplementary Table 7. Linkage disequilibrium (LD) between the multiallelic *KIR* loci. We provide here a list of all the significant LD pairs found in the eight populations studied. Only pairs with $r^2 > 0.6$ and D' > |0.6| are shown. The *p*-value of all pairs was $< 10^{-6}$.

Supplementary Table 8. Frequencies of gene-content *KIR* haplotypes found in the eight South American populations. Only haplotypes observed at least three times were included. The nomenclature of *KIR* gene-content haplotypes is according previously described (Pyo et al. 2010; Vierra-Green et al. 2012). CTBA: Brazilians of European ancestry. BrJAP: Brazilians of Japanese ancestry. ACHE: Aché. GKW: Guarani Kaiowá. GND: Guarani Ñandeva. GRC: Guarani Mbya. KIV: Kaingang from Ivaí. KRC: Kaingang from Rio das Cobras.

Supplementary Table 9. Frequencies of *HLA* alleles that code for KIR ligands (i.e. not carrier frequency). Bw4 frequencies are presented separately for the epitopes found in HLA-A or HLA-B molecules. Bw4 in HLA-B is further subdivided in Bw4I (isoleucine at position 80) and Bw4T (threonine at position 80), because this polymorphism confers differential affinity of the epitope to distinct KIR. However, Bw4T is not found in HLA-A molecules. Blank cells indicate the epitope was not found in the corresponding population. CTBA: Brazilians of European ancestry. BrJAP: Brazilians of Japanese ancestry. ACHE: Aché. GKW: Guarani Kaiowá. GND: Guarani Ñandeva.

Supplementary Table 10. Frequencies of HLA-C lineages. CTBA: Brazilians of European ancestry. BrJAP: Brazilians of Japanese ancestry. ACHE: Aché. GKW: Guarani Kaiowá. GND: Guarani Ñandeva. GRC: Guarani Mbya. KIV: Kaingang from Ivaí. KRC: Kaingang from Rio das Cobras.

Supplementary Table 11. Characterization of the study populations.
